# 2939. Hepatitis A vaccine immunogenicity in an immunocompromised population: a prospective cohort study

**DOI:** 10.1093/ofid/ofad500.178

**Published:** 2023-11-27

**Authors:** Jenny Schnyder, Hannah Garcia Garrido, Neeltje Kootstra, Martin Grobusch, Abraham Goorhuis

**Affiliations:** Amsterdam UMC - location University of Amsterdam, Amsterdam, Noord-Holland, Netherlands; Amsterdam UMC - location University of Amsterdam, Amsterdam, Noord-Holland, Netherlands; Amsterdam UMC, Location University of Amsterdam, Amsterdam, Noord-Holland, Netherlands; Amsterdam UMC, Location University of Amsterdam, Amsterdam, Noord-Holland, Netherlands; Amsterdam UMC, Location University of Amsterdam, Amsterdam, Noord-Holland, Netherlands

## Abstract

**Background:**

In the current era novel immunosuppressive therapies and improved health status of immunocompromised patients (ICPs), international travel has become more accessible for this group, putting them at risk of travel-related infections. As hepatitis A (hepA) is a common vaccine-preventable disease in travellers, guidelines recommend immunization of all travellers to endemic areas. HepA vaccination is highly immunogenic in healthy individuals, however there is uncertainty about the immunogenicity in ICPs.

**Methods:**

This prospective cohort study assessed the immunogenicity of hepA vaccination among patients living with HIV (PLWH), patients on immunosuppressive monotherapy, patients on immunosuppressive combination therapy, and controls not on immunosuppressive therapy. Participants received two hepA vaccine doses at months 0 and 6-12, or three combined vaccine hepA/B doses at months 0, 1 and 6-12. Quantitative antibody responses were measured at the first and last vaccination, 2 months after the first, and 2 and 6 months after the last vaccination (**Figure 1**). The primary outcome measure was the seroconversion rate 2 months after the last hepA vaccination, defined as the proportion of vaccinated participants with an antibody level ≥ 20 mIU/mL.

**Figure 1.**
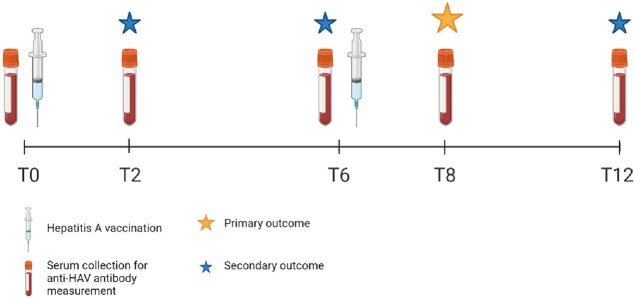
Study design. T0 = at first vaccination; T2 = 2 months after 1st vaccination; T6 = at last vaccination; T8 = two months after last vaccination; T12 = six months after last vaccination; HAV = hepatitis A virus.

**Results:**

We included 129 participants (**Table 1**). Among PLWH, patients on monotherapy, combination therapy and controls, respectively 18/32 (56%), 19/35 (54%), 11/32 (34%) and 23/25 (92%) subjects seroconverted after the first hepA vaccination and 25/26 (96%), 31/32 (97%), 23/28 (82%), 21/21 (100%) after the last hepA vaccination. Six months after the last hepA vaccination seroconversion rates were 26/26 (100%), 26/30 (87%), 21/30 (70%), 21/22 (96%), respectively (**Figures 2 and 3**).

**Table 1.**
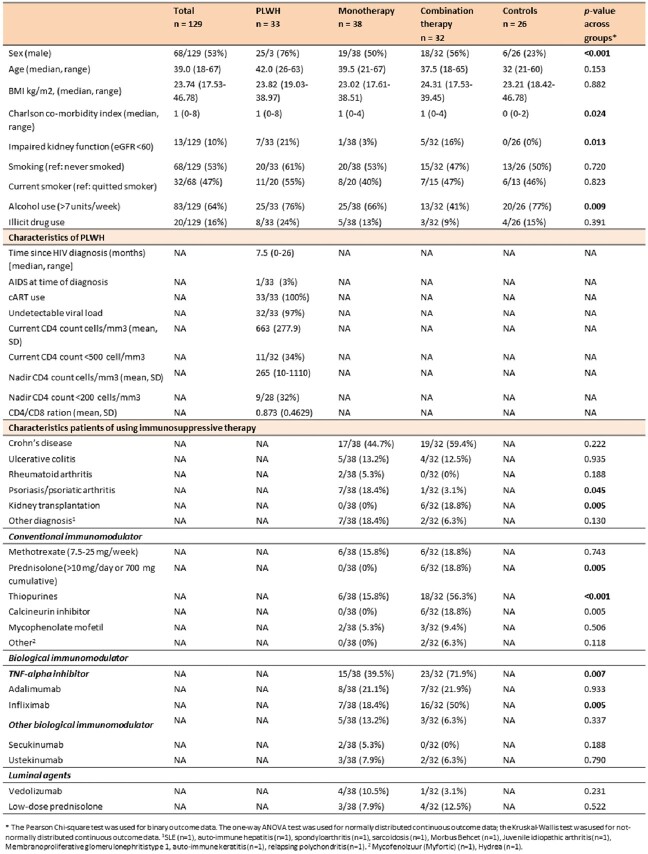
Baseline characteristics.

**Figure 2.**
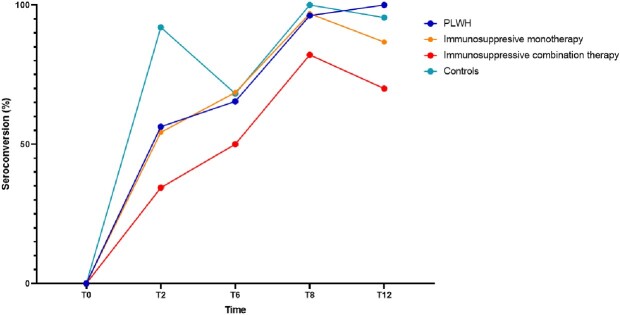
Seroconversion rates over time in immunocompromised patients and controls. T0 = at first vaccination; T2 = 2 months after 1st vaccination; T6 = at last vaccination; T8 = two months after last vaccination; T12 = six months after last vaccination, PLWH = people living with HIV.

**Figure 3.**
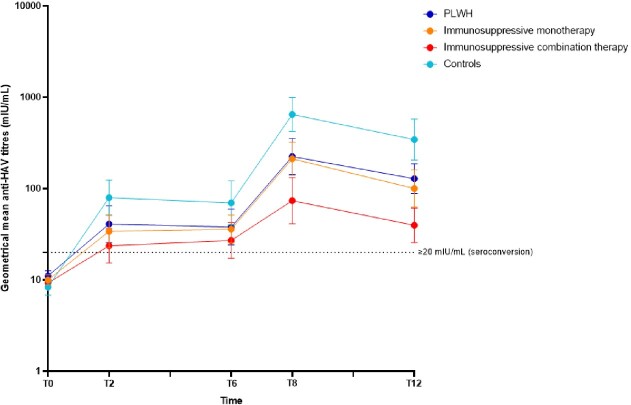
Hepatitis A antibody responses in immunocompromised patients and controls. Bars represent 95% confidence intervals. T0 = at first vaccination; T2 = 2 months after 1st vaccination; T6 = at last vaccination; T8 = two months after last vaccination; T12 = six months after last vaccination, IU = international units; PLWH = people living with HIV.

**Conclusion:**

Impaired immune responses were observed in PLWH and patients on immunosuppressive therapy after a single hepA vaccine dose, and in patients on immunosuppressive therapy 2-6 months after the last hepA vaccine dose. The implication of this finding is that serological testing in these ICPs is required before travel to endemic areas, to assess seroprotection. However, serological testing may not be necessary in virologically suppressed PLWH on cART who completed a full 2 dose hepA vaccination series.

**Disclosures:**

**All Authors**: No reported disclosures

